# Real-Time Back Surface Landmark Determination Using a Time-of-Flight Camera

**DOI:** 10.3390/s21196425

**Published:** 2021-09-26

**Authors:** Daniel Ledwoń, Marta Danch-Wierzchowska, Marcin Bugdol, Karol Bibrowicz, Tomasz Szurmik, Andrzej Myśliwiec, Andrzej W. Mitas

**Affiliations:** 1 Faculty of Biomedical Engineering, Silesian University of Technology, 41-800 Zabrze, Poland; marta.danch-wierzchowska@polsl.pl (M.D.-W.); marcin.bugdol@polsl.pl (M.B.); andrzej.mitas@polsl.pl (A.W.M.); 2 Science and Research Center of Body Posture, College of Education and Therapy in Poznań, 61-473 Poznań, Poland; bibrowicz@wp.pl; 3 Faculty of Arts and Educational Science, University of Silesia, 43-400 Cieszyn, Poland; tomasz.szurmik@us.edu.pl; 4 Laboratory of Physiotherapy and Physioprevention, Institute of Physiotherapy and Health Science, Academy of Physical Education in Katowice, 40-065 Katowice, Poland; a.mysliwiec@awf.katowice.pl

**Keywords:** anatomical landmarks, real-time detection, trunk surface metrics, physiotherapy, point cloud

## Abstract

Postural disorders, their prevention, and therapies are still growing modern problems. The currently used diagnostic methods are questionable due to the exposure to side effects (radiological methods) as well as being time-consuming and subjective (manual methods). Although the computer-aided diagnosis of posture disorders is well developed, there is still the need to improve existing solutions, search for new measurement methods, and create new algorithms for data processing. Based on point clouds from a Time-of-Flight camera, the presented method allows a non-contact, real-time detection of anatomical landmarks on the subject’s back and, thus, an objective determination of trunk surface metrics. Based on a comparison of the obtained results with the evaluation of three independent experts, the accuracy of the obtained results was confirmed. The average distance between the expert indications and method results for all landmarks was 27.73 mm. A direct comparison showed that the compared differences were statically significantly different; however, the effect was negligible. Compared with other automatic anatomical landmark detection methods, ours has a similar accuracy with the possibility of real-time analysis. The advantages of the presented method are non-invasiveness, non-contact, and the possibility of continuous observation, also during exercise. The proposed solution is another step in the general trend of objectivization in physiotherapeutic diagnostics.

## 1. Introduction

In recent years, technology development has made it possible to collect new, previously unknown types of data. This also enables the improvement of real-time analysis methods and increases accuracy, which creates new possibilities in many fields related to observing changes occurring in the human body. Real-time human state and movement monitoring is currently used in many fields, including defense, surveillance systems, entertainment, activity, and health monitoring.

Issues related to posture disorders, prevention, and therapy are still current in the public space and scientific reports. The term "posture" means the automatic maintenance of the body position in space [1]. It is also described in the literature as a process of interaction between the musculoskeletal system and the central nervous system that maintains the body in a state of musculoskeletal equilibrium under static and dynamic conditions [2,3]. Due to this, the body is also protected from injury or progressive deformity. Biomechanical, neurophysiological, and emotional factors play an essential role in maintaining posture [4,5].

Widely described theoretical and clinical research has considered the tri-planar human body structure and functioning [6]. The most common postural disorders in the sagittal plane include lordotic, kyphotic, kyphotic-lordotic, and flat-back posture [7]. Consequently, when untreated, they can lead to severe medical conditions and pain. The magnitude of postural disorders and their effects are described in the literature. It is estimated that over 80% of the American population requires medical intervention for back pain [8].

Screening shows that more than 50% of children and adolescents exhibit postural abnormalities, with 10% of this group at risk for scoliosis or other progressive spinal deformities [9]. Postural abnormalities result from multifactorial physical and psychosocial, genetic, environmental, emotional, and socioeconomic changes. They can make it difficult to perform daily activities and cause degenerative diseases, including spinal disease [10,11]. Lifestyle, exercise habits, rest and sleep hygiene are the most critical determinants of human posture [3].

Assessment of human posture is one of the standard procedures used by physicians, physiotherapists, or physical culture specialists. This identifies postural deviations and monitors disease progression or the effects of therapy. The gold standard in the assessment of posture and spinal deformities is the radiological examination [12]. The procedure’s high financial costs, difficult availability, and impact on the subjects’ health (invasiveness through radiation exposure) make its use limited. Skeletal deformities directly affect postural appearance so that specialists can perform an initial qualitative assessment of posture based on general observation.

Non-invasive tools are now also available for quantitative assessment based on trunk surface topography [13]. The metrics used in clinical practice are usually determined by the values of the angles of individual curvatures in three planes using inclinometers or gravity goniometers. The second type of trunk surface metrics based on the topography of the back surface includes indices determined based on the mutual arrangement of anatomical landmarks in the frontal plane. Due to their high availability and low cost, these quantitative parameters are primarily used to implement screening and evaluate the effects of therapy between treatment sessions. A limitation of the non-invasive methods is the need for direct or indirect (on the photo) determination of marker positions by an expert, which increases the testing time and differentiates results due to inter-rater reliability. The confirmed posture assessment accuracy makes them a basis for developing new diagnostic support tools ensuring accuracy and repeatability [14,15,16]. The development of modern approaches also creates new possibilities in performing postural assessments while exercising or during activities of daily living.

Advances in technology contribute to the development of new tools to aid in the diagnosis of postural malformations. These include attempts to augment or replace existing tools with modern technologies. The most recent trend in diagnosis support is to use sensors and cameras built into smartphones [17]. Modern posture measurement tools are sophisticated systems that use a variety of measurement techniques. One well-known approach involves the use of single photographs to carry out photogrammetric measurements [15]. Techniques to obtain additional depth information include structured light imaging [18,19], 3D scanners, multi-camera systems, and depth cameras, including the Kinect system [20].

Currently, developed techniques allow obtaining whole-body scans. However, depending on the technique chosen, limitations arise regarding the high costs and lack of mobility in multi-camera systems or lack of data recording over time in 3D scanners. Systems using a single camera with a depth sensor allow for back surface mapping. The obtained data provide an evaluation of the posture in three planes. Due to the data analysis algorithms used, posture assessment support systems can be based on the parameters determined from the point cloud deformation [21] or based on known trunk surface metrics determined from the location of anatomical landmarks [13]. In the second case, if the system is not equipped with an automatic detection algorithm, the expert must mark the points (direct on the subject’s body or the obtained data). Another technology trend currently under development among many academic teams, is wearable sensors [22,23,24,25].

Although the computer-aided diagnosis of posture disorders is well developed, there is still a need to develop existing solutions, search for new measurement methods and create new algorithms for processing [16]. In addition to high accuracy, the low cost of the system, and examination, it is necessary to evaluate posture not only in static conditions but also regarding variability during movement [26].

This work aims to develop a method for automatically detecting selected anatomical landmarks on the human back surface based on point clouds collected with a Time-of-Flight (ToF) camera. The fundamental goal of the created algorithm is to work in real-time with a sampling rate that allows for monitoring of the subject’s posture during therapeutic exercises in a dedicated device, Disc4Spine (D4S).

## 2. Materials and Methods

### 2.1. Acquisition Setup

The presented acquisition setup is an integral part of a larger, original device, called Disc4Spine, which is equipped with various measuring systems for assessing the patient’s condition during diagnosis and therapeutic procedures of postural defects [27].

The D4S device uses an SR4000 camera (MESA Imaging 107 AG, Switzerland) capable of acquiring depth images in *x*, *y*, *z* coordinate matrices containing 144 × 176 measurement points. The camera allows point cloud acquisition at a frequency of 30 Hz.

**Figure 1:**
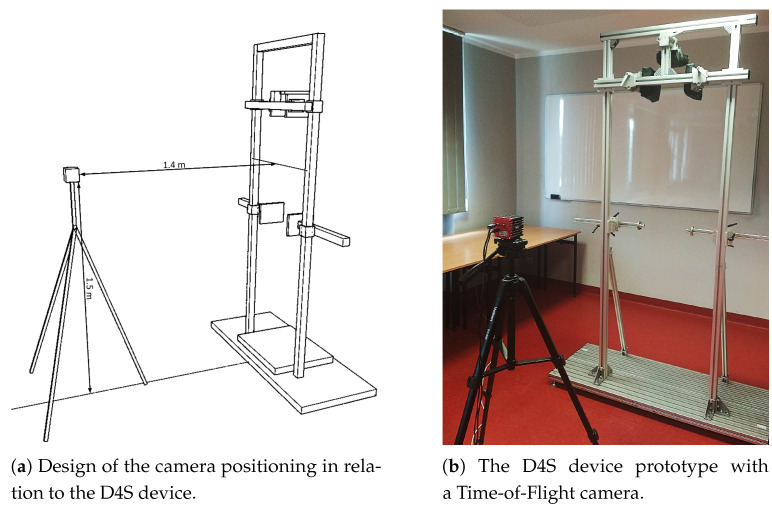
The concept of the D4S system module for monitoring exercises in the standing position.

The camera was placed on a tripod 1.4 m from the D4S module at the height of 1.5 m (Figure 1). It allowed observation of the full D4S frame width and an area from approximately 1 m to 2 m height. The resulting point clouds contained the full range of the subject’s back, considering the variation in body length, without the requirement to manipulate the camera position. The camera coordinate system corresponds to the anatomical axes of the subject as follows:

*x*—frontal axis (from left to right),
*y*—longitudinal axis (from bottom to top), and
*z*—sagittal axis (distance from the camera).

### 2.2. Research Protocol

The control group consisted of 53 students (24 female, 29 male) of the Jerzy Kukuczka Academy of Physical Education in Katowice aged from 19 to 26 years. The participants voluntarily responded to the invitation to participate in the study and gave their written informed consent. The physiotherapy expert had listed exclusion criteria, i.e., pain during movement tasks, mobility limitations, and problems in understanding the commands. All the subjects were measured by a qualified anthropologist, and the general description of the group is presented in Table 1. The inclusion criteria specified by physiotherapist experts to meet all the project assumptions [28] were:

1. age in the range of 19–29 years (young adults),
2. be able to provide informed consent for the study,
3. without any dysfunction in auditory processing or significant visual impairment,
4. declaring physical activity in the form of various sports exercises,
5. a lack of locomotor dysfunctions that may affect the measurements due to pain or limitations of mobility range, and
6. that they are not in psychiatric treatment or crisis.

One step of the experimental protocol was to perform consecutive anterior and posterior pelvic tilt in standing position for 60 s at the frequency around one sequence (anterior–posterior) per second. The exercise was recorded continuously with a ToF camera, and the recordings were saved as a series of the subject’s body point clouds. We chose the pelvic tilt from all different exercises provided by the subject in the D4S device during the research because it allows obtaining point clouds with different poses in the sagittal plane, not only the habitual position.

### 2.3. Point Clouds Preprocessing

**Figure 2:**
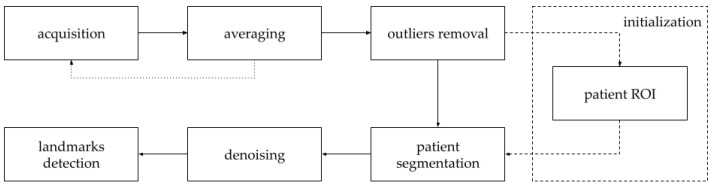
General scheme of the point cloud preprocessing. The averaging step aims to increase the mapping precision of the subject’s silhouette. Subsequent steps allow the reduction of additional elements and to denoise the resulting subject’s point cloud.

The depth range was limited to 2 m in the acquisition process to reduce the number of additional scene elements. A high noise level characterizes data collected from a single acquisition. The pre-processing is aimed at reducing the noise and automatically determining the region of interest related to the silhouette of the examined person (Figure 2). At the stage of acquisition, noise reduction is carried out by averaging the coordinates of successively obtained matrices [29]. Unfortunately, such an approach causes the time resolution to be decreased.

**Figure 3:**
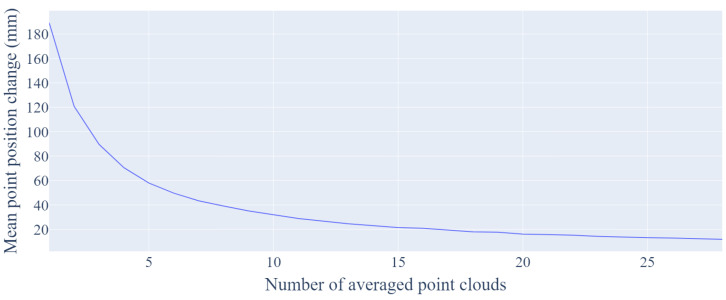
The mean point position change concerning to the number of averaged point clouds. The Euclidean distance was calculated for the corresponding points in the coordinate matrices obtained by averaging the successive N and N-1 point clouds.

As the point detection algorithm has to work in real-time, and the minimum frequency, which is necessary to ensure the correct operation of other components of the planned system (e.g., analysis of the correctness and effectiveness of the performed exercises, the gaming module), has to be assured, the number of averaged clouds was set to 10. In Figure 3, a plot of the mean change of the points position as a result of averaging successive point clouds is presented. In order to reduce the noise level, the outlier detection was performed based on the neighborhood criterion adjusted to the measurement conditions. Ultimately, a point is treated as an outlier if the number of points in a sphere with a radius of 5 cm is less than 20.

The algorithm for the automatic determination of the region of interest, which is a part of the D4S device, is run once at the beginning of each measurement session. In the first step, the cloud is divided into the left and right sides in relation to the position of the centroid. Then, the bottom of each cloud is selected—10 cm above the lowest point. In the obtained fragment of the cloud, a part of the subject’s lower limb and the lower part of the side element of the device’s structure are present.

**Figure 4:**
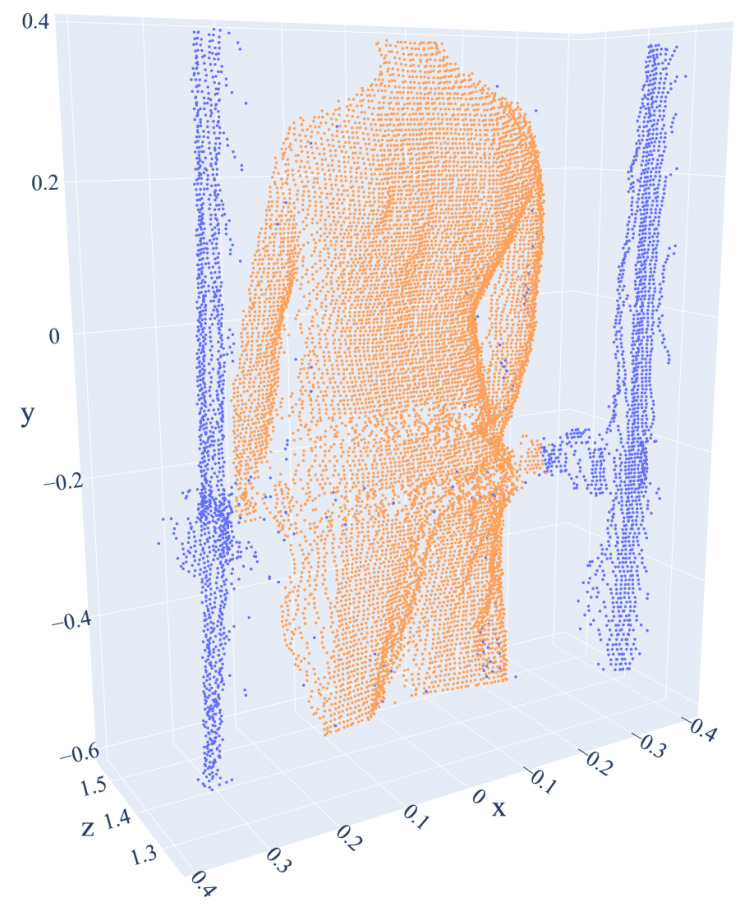
Result of point cloud preprocessing. The analyzed subject’s silhouette is marked in orange. The remaining points that are part of the device, and the points removed due to noise reduction (outliers) are blue.

Using DBSCAN clustering [30] (epsilon = 0.03), both objects were separated, and the device elements were automatically segmented. As a result, the *x* coordinates of the analyzed cloud were limited to the range defining the inside of the device. The computed range was used in subsequent point cloud acquisitions to segment the subject’s silhouette. The subject’s point cloud was then denoised using the neighborhood criterion (radius 3 cm and number of neighbors: 10). The results of the input data preprocessing are presented in Figure 4.

The preprocessing algorithm was adapted to work as an integral part of the D4S system. For this reason, unless otherwise stated, all thresholds were empirically selected to maximize the real-time point cloud processing efficiency.

### 2.4. Landmarks Detection

The algorithm for detecting particular landmarks is based directly on the properties of the individual cloud fragments. Some of the analyzed fragments are connected with other, previously detected ones. The detection of points indicating the position of the axilla ($A_{L}$ and $A_{R}$) begins with localizing the auxiliary points marking the boundary between the trunk and the upper limbs. In order to detect them, a part of the silhouette is selected at the position of the centroid. Next, the DBSCAN clustering allows the torso and arms to be separated, and the range of torso *x* coordinate can be approximated. The cloud fragment, separately for the left and right sides, selected from the obtained auxiliary point is approximated using linear least squares regression.

The points located under the obtained plane, taking into account the distance threshold form a coherent object. The highest point of this object determines the location of the axilla. The shoulders points ($S_{L}$ and $S_{R}$) are the highest points in the belt of points defined by the *x*-coordinate of the corresponding axilla. The frontal plane location of the vertebra prominens at the level of the cervicothoracic junction (C7-T1) is the midpoint of the shoulder line. Mapping this location on the silhouette point cloud is done by finding the point closest to the center of the line and omitting the *z* axis.

The cervicothoracic junction point (C7-T1) indicates the position of the spine line. On this basis, the location of the landmarks used to determine the value of the trunk surface metrics in the sagittal plane is determined: thoracic kyphosis (TK), lumbar lordosis (LL), and the sacral base (estimating the location of S1). Points located on the spine line are approximated with polynomials in the yz plane. Local extremes of the approximation indicate the *y* coordinate of thoracic kyphosis (TK) and lumbar lordosis (LL). The lowest point of inflection is an approximation of the location of the sacral base (S1). Mapping points to the cloud surface is performed by selecting a fragment of the cloud in relation to the found *y* position, specifying the *x* coordinate in the center of the chosen area, and finding the nearest neighbor.

### 2.5. Method Validation

An 80-s fragment was selected for each subject containing 10 s of preparation, 60 s of the exercise, and 10 s of rest. Next, from each fragment, eight frames were selected at intervals of 10 s and saved in separate files. This allowed obtaining data from various postures during the exercise—intermediate states between anterior and posterior pelvic tilt and the habitual position.

The resulting validation dataset was created based on 424 clouds from 53 subjects in eight different body positions. On selected frames, three physiotherapists marked anatomical landmarks using specially prepared software. The software enabled the marking of points in three-dimensional space with the mouse pointer. It was equipped with basic functions for the comfortable manipulation of a point cloud (rotation, panning, and zoom).

After loading a single point cloud of a given participant, the expert indicated the locations of all 10 landmarks. To facilitate the work, the software allowed the expert to choose any order of pointing and to modify previous pointing when needed. The experts had not seen the landmark locations selected by other experts and the method results before the procedure. They were trained in advance to use the software on a sample dataset. The experts were physiotherapists with over 25 years of experience in running medical practices, academics conducting classes, and instructors from courses in the treatment of postural defects.

The obtained data were used to assess the differences between the expert point localizations and to validate the proposed method. In the first stage of the analysis, the absolute error for all indications of experts and the method was determined in the xy system, following the equation $\Delta L\left(x,y\right)=L\left(x,y\right)-L_{0}\left(x,y\right)$. The expected value $L_{0}\left(x,y\right)$ was estimated by averaging the indications of three experts for a given landmark L(x,y) in the analyzed point cloud. From the obtained values, the mean absolute error and standard deviation of absolute error were determined for each expert and method. In order to eliminate the influence of the coarse error, the outlier removing procedure for the detection method results was performed.

All ΔL(x,y) for each landmark were transformed into the principal component space. The interquartile range rule in the principal component space was used to define the point as an outlier, independently for both components, removing values higher than $Q_{3}+1.5IQR$. After removing outliers, the remaining points were transformed back into the (*x*, *y*) space of the subject coordinate system. The second validation stage included comparing the experts’ indications and comparing the method results with each of the experts independently.

For each point, for each case, the Euclidean distance between the two locations indicated by the compared experts was determined. For the obtained distances, the average distance [31] value was calculated. For the method, the average value of the distance compared to each of the experts was calculated separately. The average distance value of all performed method vs. expert comparisons was also calculated for each landmark.

Experiments were performed on a workstation running a 64-bit Windows 10 operating system with an Intel Core i7-8750H CPU, 32 GB DDR4 RAM. The described methodology was implemented in Python 3.7 with NumPy [32] and SciPy [33] libraries for basic matrix operations and linear regression. The DBSCAN and outlier removal algorithms came from the Open3D library [34].

### 2.6. Statistical Analysis

In order to evaluate the statistical significance of the differences between the proposed method and experts the Friedman test was performed for all six groups (each expert vs. the proposed method—three groups and each expert vs. each expert—three groups). The Friedman test was chosen, since the assumptions for ANOVA were not fulfilled. Its effect size was calculated as the Kendall’s *W* value, whose interpretation is similar to Cohen’s *d*.

## 3. Results

The point clouds obtained as a result of the preprocessing and segmentation of the subject’s silhouette were visually verified based on the entire 80-s fragments of recordings for all 53 subjects. No errors were reported in the proposed method. In every case, the region of interest for the segmentation of the cloud fragment used as the input for the detection algorithm was correctly identified. The applied denoising method contributed to reducing outliers while maintaining all the relevant information needed in the subsequent stages of processing.

**Figure 5:**
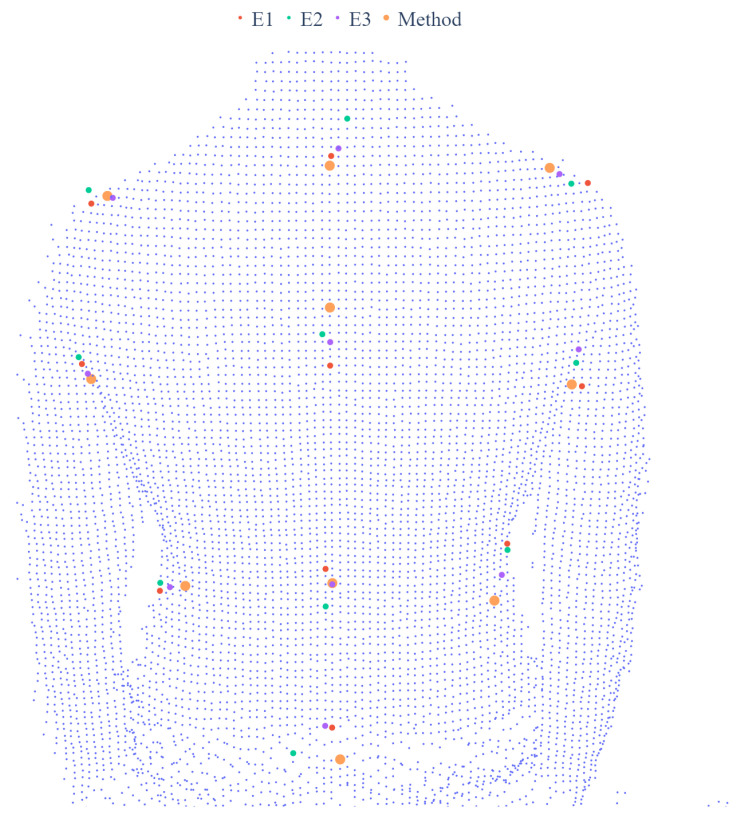
The result of detection, for one frame, of anatomical landmarks with the described method compared with the expert indications.

The average time of processing of one frame was 0.1 s. However, due to the frame averaging process, the algorithm could run in real-time at a sampling rate of 3 fps. An example of the result of the proposed landmark detection method compared with the indications of experts is presented in Figure 5.

**Figure 6:**
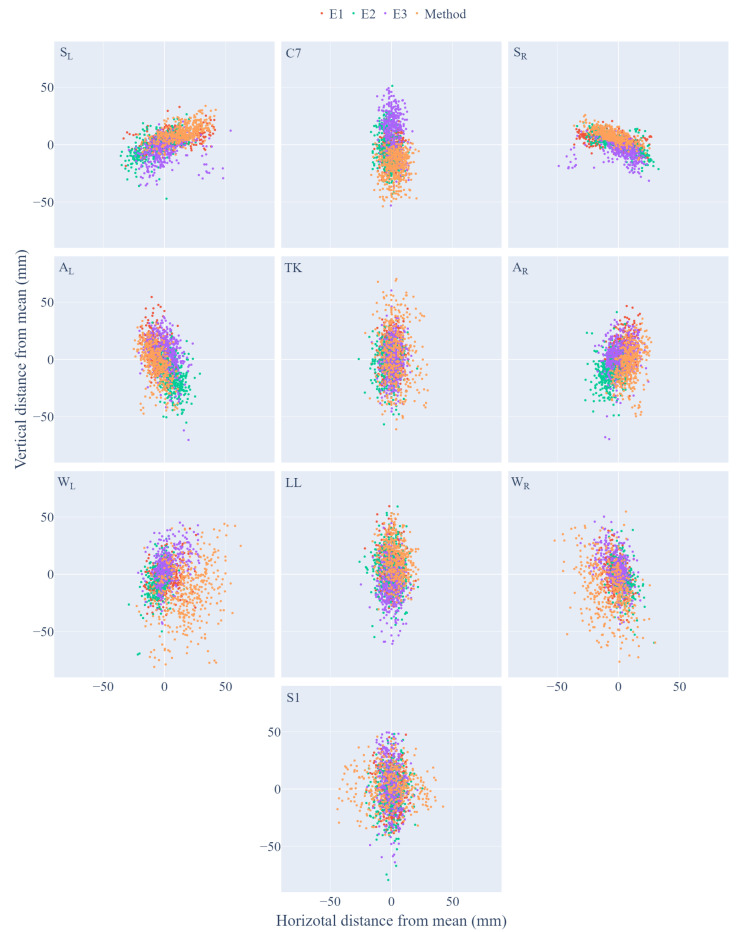
Visualization of the relative error for individual landmarks, after removing outliers; the point markers have been transformed in such a way that the expected value for experts is the origin of the coordinate system.

The absolute error values for the method and each expert calculated for 424 selected clouds are presented in Figure 6. The results obtained for the method in most cases are located within the area of experts’ recommendations. The numerical values for the mean absolute error and standard deviation of the absolute error are in Table 2 and Table 3, respectively.

The mean absolute error of the experts for all landmarks apart from the shoulders was less than 6 mm in the frontal axis (*x*) and less than 10 mm in the vertical axis (*y*). The standard deviation of the absolute error for experts, except shoulders ($S_{L}$, $S_{R}$), was lower than 10 mm in the frontal axis (*x*) and lower than 20 mm in the vertical axis (*y*). The method gave results shifted in the medial and cranial direction for shoulders landmarks ($S_{L}$, $S_{R}$) and in a lateral and caudal direction for axilla landmarks ($A_{L}$, $A_{R}$).

The standard error deviations for these points are comparable when the individual experts are compared with each other. The waist indentation points ($W_{L}$, $W_{R}$) had the highest value of the mean absolute error and its deviation in both directions. This value was not reduced after removing the outliers, but the mean did not exceed 20 mm in both cases. However, the spread of the error value of the method was more than 10 mm higher than in the case of expert error. Landmarks located in the spine axis were detected with the average error close to the experts’ error in the frontal axis (*x*).

In the vertical axis, the C7-T1 point was detected by the method, on average, 20 mm lower than the expected value, and the LL point was 13 mm higher. The method error deviation in the frontal axis (*x*) was about 10 mm higher than the expert error deviation for S1. In the vertical axis (*y*), the TK spread was about 10 mm greater than the expert error spread.

The outlier removal eliminated points that significantly deviated from the main cluster of individual error values. In some cases, this improved the value of the error evaluation metric. Table 4 presents the number of observations considered as outliers for individual landmarks along with their percentage share in the entire analyzed set.

Table 5 shows the average distance of the points located between the pairs of experts and the method. The discrepancies between independent experts were, in most cases, less than 30 mm. The average distance value between experts and method reached the highest value for the waist points. The obtained average distances were more than 10 mm greater than the discrepancy between the experts. For the *A* and *S* points, the distances obtained for the method were close to the discrepancy between experts. Similarly, for points in the spine axis, except for TK, the method showed slightly greater discrepancies than the experts’ indications (by about 5 mm).

The *p* value was lower than 0.05 in all cases (Table 6), which means that there was statistical significance in the difference between the medians in the analyzed groups. However, the *W* values were below 0.1 in most cases, which means that the effect was negligibly small, and, in three cases, it was lower than 0.3, which should be interpreted as a small effect. The statistical significance of the null-hypothesis significance testing is the product of several factors: the “true” effect size in the population, the size of the sample used, and the alpha (*p*) level selected [35].

With a sufficiently large sample, a statistical test will almost always demonstrate a significant difference, unless there is no effect at all [36]. The obtained *W* values lead to the conclusions that the statistical significance results from the large groups sizes. Effect sizes are resistant to sample size influence and, thus, provide a truer measure of the magnitude of effect between variables [35] and are the main finding of a quantitative study [36]. Therefore, the differences between the compared groups can be interpreted as not of practical significance. When the sample size was reduced to 50, there were two types of outcomes for the eight examined points: (1) the Friedman test was not significant (p>0.05) or (2) the Friedman test was significant, but the effect size was very small.

## 4. Discussion

The presented results show that the proposed method provided comparable results to the manual procedure. Since the method is able to perform in real-time, the advantage in comparison to one-time manual assessment is immense. Based on obtained results, one can compare the tendencies of individual experts resulting from experience and subjective assessment, e.g., expert E2 marked the axilla clearly lower than expert E3. On the other hand, expert E1 marked the shoulders higher than the other two experts.

Marking anatomical landmarks, especially in the spatial image and not physically on the subject, is burdened with a certain cognitive error. The accuracy of the determined landmark is influenced by the characteristics of the subject’s posture and, particularly, the level of fat tissue. When physically marking points on the subject’s body, the therapist has the opportunity to assess both visually and by palpating, which certainly increases the accuracy. However, the observation of markers marked on the skin introduces a different kind of error related to the displacement of the skin in relation to the bone structures inside the body, which are the subject of observation [13].

The proposed methodology for outlier removal reduced incorrect detections in order to compare the scatter of automatically detected points with the indications of experts. This process contributed to the reduction of the standard deviation in at least one direction while not affecting the location of the mean. From the other hand, when the outliers for the axilla and the C7-T1 point were removed, the difference of the expected value increased. This is due to the general tendency of the method to mark these anatomical landmarks lower than the experts. Hence, the outliers appeared higher than usual and brought the overall trend closer to the expert values. The determination of the waistline both by the experts and the method was burdened with a high error due to deformations caused by clothes (volunteers were wearing sports pants) and fat tissue, the folding of which made it impossible to locate anatomical landmarks unequivocally.

As a result of the applied outlier reduction criterion, at most 8% of the points for each landmark were removed from the first stage of the analysis (comparison of the mean and deviations). The gross errors were not related to any specific subject. They occurred in single clouds for different subjects. The cause of the errors was the temporary atypical position of the posture or upper limbs, which could have resulted in an incorrect indication of the search areas at the first stages of the algorithm.

The greater number of errors for the points on the right side ($A_{R}$, $S_{R}$) may suggest misalignments in the camera positioning. Incorrect point detection in the first steps of the algorithm contributed to errors in the localization of other points due to the relations between them. In further works, the wrong detections can be reduced by using real-time filtration or tracking algorithms, due to which, the search area will be limited, and the confidence metric will be proposed for each detection.

The mean values of the distance between the method and all experts indicated the greatest discrepancy in the case of the waistline (mean of 36.35 mm for both sides). The values for the symmetrical landmarks of the axilla and shoulders were 25.11 mm and 21.53 mm, respectively. Kozbial et al. [31] proposed an algorithm to detect certain anatomical landmarks, including those mentioned earlier, over whole-body scans using a convolutional neural network. The highest value of the arithmetic means between the method and manually-marked points was achieved for the lowest rib point of 29.7 and 37.5 mm in the two proposed methods. The detection efficiency of this landmark can be related to the waistline point due to its proximity. The achieved values are close. Similar conclusions can be drawn from comparing the results for the axilla (21.3 mm) and the shoulders (15.8 mm). The method proposed by us is based on back surface point clouds in the form of scattered points, which, unlike whole-body scans, may result in a less accurate representation of the posture but enable the creation of a real-time solution.

In most cases, the detection error between the presented method and the experts did not differ from the error in assessment between experts. A direct comparison of the distances of the indicated points between experts and the method showed that the compared differences were statically significantly different; however, the effect was negligible. The lack of location of the landmarks marked by experts directly on the subject’s body limited the results obtained. However, because the measurements were performed under dynamic conditions, it was impossible to determine the anatomical structure positions in variable poses. Marking markers on the skin, in this case, would also be subject to error due to changes in the location of anatomical structures under the skin.

The method reflects in implementation the experts’ localization technique. In contrast to the machine learning approach, the proposed method does not rely on expert indications in the training process. Instead, it employs mathematical methods to calculate the functions extremes determined based on selected fragments of recorded subject clouds. The analysis of other approaches described in the literature and evaluation of discrepancies between experts gives grounds for the conclusion that the proposed method offers accuracy on an acceptable level [37].

The variation in body size and posture type in the study group further confirmed the results’ reliability. The young adult group is representative of patients undergoing treatment for postural defects. Further work using the proposed method can be directed toward diagnosing and assessing the degree of deformity in specific postural defects. This will require a study on an appropriately selected research group.

Continued use of the system in patient therapy will allow adjustment of the device and detection algorithm to further minimize the errors that occur. Proper patient involvement in the technical aspects of therapy in the proposed system is an important challenge that the researchers are fully aware of.

The processing time achieved on a middle-class workstation shows that the algorithm can be used for data obtained with a higher sampling frequency. As the algorithm is based only on raw scatter point locations, it is possible to test it using various techniques of depth image acquisition.

Accurate depth detection with Time-of-Flight cameras requires appropriate parameter adjustments to achieve a satisfactory signal-to-noise ratio while maintaining the correct sampling rate. It is also important to consider the properties (i.e., the color, shape, and material) of objects in the camera’s field of view: objects in the background and objects too close to the camera. Even if these objects are removed with a signal processing algorithm, they can introduce depth distortion during acquisition in other point cloud regions [38].

Therefore, using a ToF camera in a D4S system requires all system components to be set up correctly and adjusted. The proposed method is independent of the point cloud acquisition method; however, changing the spatial resolution may require tuning the parameters related to point cloud preprocessing. A more significant number of measurement points may result in an increased computation time, but the averaging process can be omitted in this case because of the more accurate representation at the acquisition stage.

The use of the Time-of-Flight camera in the developed system for supporting the diagnosis and therapy of posture defects provides the possibility of obtaining relevant data in real-time while ensuring portability and relatively low costs compared to systems for whole-body scans. The collected data in the form of scatter point clouds were used to effectively detect anatomical landmarks and observe the variability of their position during therapy of mild posture defects. This is a promising introduction to the development of a posture correction support system that meets all the current requirements [16].

Physiotherapeutic exercises usually take place in a limited space. Hence, adopting the proposed algorithm in any working conditions should not cause a problem as long as it is possible to identify the patient’s pose in space, and the characteristics of the exercises enable the observation of the back surface, such as exercises performed on therapeutic treadmills or stabilometric platforms. Further improvements using point tracking and machine learning methods will be included in our further investigations.

## 5. Conclusions

This research proved the possibility to create an accurate and fast anatomical landmark detection method based on back surface point clouds from a Time-of-Fight camera. The proposed method was evaluated with manual markers from three independent experts, thus, strengthening the evidence of achieved results. To a significant extent, the proposed solution allowed for an objective and harmless objective assessment of both long- and short-term changes during the treatment process.

## Figures and Tables

**Table 1:** General description of the research group.

	Female	Male	All
Body weight (kg)	65.9 ± 18.9	85.2 ± 9.4	75.9 ± 17.5
Body height (cm)	166.5 ± 6.8	180.8 ± 7.1	174.2 ± 10.0
Shoulder width (cm)	36.6 ± 1.9	40.0 ± 2.2	38.4 ± 2.7
Chest width (cm)	25.3 ± 3.0	28.6 ± 1.8	27.1 ± 3.0
Hip width (cm)	28.0 ± 2.2	28.8 ± 1.5	28.5 ± 1.9
Bust size (cm)	77.0 ± 9.3	88.8 ± 7.1	83.4 ± 10.0
Waist size (cm)	73.1 ± 11.2	80.9 ± 6.9	77.3 ± 9.8

**Table 2:** The mean absolute error of points selected by a given expert in relation to the expected value estimated by averaging the indications of three experts. The table on the left shows the value in the frontal axis (*x*), and, on the right, the vertical axis (*y*). Values expressed in mm.

Landmark	$E1_{x}$	$E2_{x}$	$E3_{x}$	$M_{x}$	$M_{\mathrm{outx}}$	$E1_{y}$	$E2_{y}$	$E3_{y}$	$M_{y}$	$M_{\mathrm{outy}}$
$A_{L}$	−5.4	5.1	0.2	−6.7	−6.5	6.0	−12.2	6.2	−6.1	−3.6
$A_{R}$	2.9	−5.1	2.1	8.6	9.0	4.9	−9.3	4.3	−7.7	−4.1
$S_{L}$	3.0	−3.8	0.8	14.1	14.1	3.5	0.2	−3.7	10.8	10.4
$S_{R}$	−0.8	−1.2	2.0	−4.8	−4.2	2.3	1.7	−4.0	7.5	7.5
$W_{L}$	0.7	−3.2	2.5	18.4	18.1	−2.1	−3.9	6.0	−14.1	−15.4
$W_{R}$	−1.2	2.5	−1.3	−8.9	−7.7	−1.8	−2.4	4.2	−11.0	−12.4
C7-T1	0.4	−2.1	1.7	2.3	2.9	−4.9	−4.4	9.3	−19.5	−19.1
TK	0.2	−1.8	1.6	5.6	3.7	3.8	−2.8	−1.0	3.5	4.3
LL	0.3	−0.6	0.2	3.5	3.5	3.7	4.8	−8.4	15.2	12.9
S1	0.7	−0.1	−0.5	−1.7	−0.9	−1.5	−2.5	4.0	4.2	2.0

**Table 3:** Standard deviation of the absolute error of points selected by a given expert in relation to the expected value estimated by averaging the indications of three experts. The table on the left shows the value in the frontal axis (*x*), and, on the right, the vertical axis (*y*). Values expressed in mm.

Landmark	$E1_{x}$	$E2_{x}$	$E3_{x}$	$M_{x}$	$M_{\mathrm{outx}}$	$E1_{y}$	$E2_{y}$	$E3_{y}$	$M_{y}$	$M_{\mathrm{outy}}$
$A_{L}$	5.6	7.6	8.3	7.5	7.3	12.4	14.4	14.7	19.0	15.0
$A_{R}$	5.8	7.6	8.0	18.4	6.8	12.4	12.2	13.1	24.0	14.9
$S_{L}$	14.2	12.6	12.8	12.9	12.8	6.6	9.2	10.2	8.0	7.4
$S_{R}$	13.3	13.8	11.6	17.0	10.8	5.3	7.5	7.8	11.2	5.7
$W_{L}$	7.4	5.5	8.4	18.7	15.8	10.8	12.1	13.7	32.2	24.2
$W_{R}$	5.8	5.2	7.3	19.6	13.5	11.7	12.4	14.2	31.6	23.3
C7-T1	4.7	5.3	5.1	9.6	7.0	9.8	14.3	18.1	13.2	11.8
TK	5.1	6.2	5.0	12.5	8.9	14.4	14.2	16.3	26.0	25.5
LL	5.6	7.1	5.8	11.1	7.3	15.7	16.7	16.2	25.7	15.7
S1	5.9	7.0	6.1	18.1	16.5	16.9	17.9	20.5	31.4	16.3

**Table 4:** The number of removed outliers as a result of the interquartile range method employed in the principal component space.

Landmark	Outliers NumberM (Percentile of Observations)
$A_{L}$	20 (5%)
$A_{R}$	33 (8%)
$S_{L}$	10 (2%)
$S_{R}$	30 (7%)
$W_{L}$	25 (6%)
$W_{R}$	25 (6%)
C7-T1	12 (3%)
TK	30 (7%)
LL	23 (5%)
S1	26 (6%)

**Table 5:** The average distance between the points determined by experts and the proposed method, expressed in millimeters. Experts are abbreviated E1–3, and the method is abbreviated with the symbol M. The last column shows the average distance from all methods vs. expert comparisons.

Landmark	E1	E2	E3	M	M	M	M
	E2	E3	E1	E1	E3	E3	All
$A_{L}$	27.36	31.65	22.21	20.02	26.58	25.23	23.94
$A_{R}$	23.95	26.54	21.07	22.35	29.01	27.47	26.28
$S_{L}$	20.61	23.89	24.52	18.59	25.58	26.64	23.60
$S_{R}$	22.04	22.16	19.90	15.88	20.55	22.03	19.49
$W_{L}$	16.54	22.24	22.71	36.25	39.58	42.43	39.42
$W_{R}$	17.00	21.09	21.37	30.72	33.13	36.01	33.29
C7-T1	16.02	31.17	25.94	19.94	23.90	31.10	24.98
TK	22.71	24.43	24.61	24.54	29.95	30.82	28.44
LL	25.73	28.26	25.00	21.24	27.91	32.03	27.06
S1	25.22	31.20	28.82	28.27	31.63	31.08	30.32

**Table 6:** The results of the statistical analysis of the differences between the proposed method and experts using the Friedman test with the effect size calculated as Kendall’s W value.

Landmark	W	Effect	*p*
$A_{L}$	0.08	negligibly small	<0.001
$A_{R}$	0.05	negligibly small	<0.001
$S_{L}$	0.04	negligibly small	<0.001
$S_{R}$	0.04	negligibly small	<0.001
$W_{L}$	0.29	small	<0.001
$W_{R}$	0.15	small	<0.001
C7-T1	0.13	small	<0.001
TK	0.04	negligibly small	<0.001
LL	0.04	negligibly small	<0.001
S1	0.01	negligibly small	<0.001

## Data Availability

The data could be provided on request after contact with the corresponding author.

## Abbreviations

The following abbreviations are used in this manuscript:

ToF	Time-of-Flight
$A_{L}$, $A_{R}$	axilla left and right, respectively
$S_{L}$, $S_{R}$	shoulder left and right, respectively
$W_{L}$, $W_{R}$	waistline left and right, respectively
C7-T1	cervicothoracic junction
TK	thoracic kyphosis
LL	lumbar lordosis
S1	sacral base
